# Cognitive decline in elderly medical and surgical inpatients

**DOI:** 10.4103/0019-5545.55954

**Published:** 2005

**Authors:** Srinivasan N. Tirupati, Rebecca N. Punitha

**Affiliations:** * Conjoint Associate, Professor Faculty of Health, The University of Newcastle, Hunter Area Mental Health, Newcastle, Australia; ** Diplomate in National Board Trainee in Family Health, Dr Rangarajan Memorial Hospital, Sundaram Medical Foundation, Chennai, India

**Keywords:** Cognitive decline, elderly patients

## Abstract

**Background:**

Impairment in cognitive function increases with age.

**Aim:**

To study the prevalence of cognitive decline in inpatients ≥60 years of age.

**Methods:**

One hundred and thirty patients (85 men and 45 women), admitted to a community general hospital for medical or surgical treatment, were selected. The Mini Mental State Examination (MMSE) was used to identify subjects with cognitive dysfunction. Patients were categorized as having cognitive decline or normal cognition. The Global Rating of Memory Decline (GRMD) and Global Rating of Intellectual Decline (GRID) scales were used to assess the decline in memory, thinking and reasoning ability.

**Results:**

Cognitive decline was diagnosed in 54 subjects (41.5%). Significantly more patients ≥70 years of age had cognitive decline compared to patients ≤70 years of age. On the GRMD, 71 patients had subjective decline in memory, 62 of them reported that the decline interfered with their daily life. On GRID, subjective decline in intellectual function was found in 91 patients, with 55 reporting that the decline interfered with their daily lives.

**Conclusion:**

Patients ≥70 years of age with an acute medical problem are the most likely to have cognitive problems.

## INTRODUCTION

Impairment in cognitive function increases with age.^1^ It has been seen that nearly 50% of people admitted in the medical and surgical wards of a general hospital are over 65 years of age.^2^ Delirium and dementia are the two common syndromes of general cognitive decline. Delirium occurs in 10%–61% and dementia in 14%–61% of elderly inpatients in general hospitals.^2^–^5^ They were associated with an increased morbidity and mortality, length of hospitalization, cost of care and caregiver stress.^3^–^5^ Medical teams often fail to diagnose cognitive decline in elderly inpatients. Inouye reports that the diagnosis of cognitive decline in the elderly was missed at rates as high as 77% in the general hospital setting.^4^

This paper reports on the prevalence of cognitive decline in patients ≥60 years of age admitted to a community general hospital in Chennai, India for medical or surgical treatment. The clinical and demographic variables associated with cognitive decline are also reported.

## METHODS

We selected 130 patients comprising 85 men and 45 women for the study. Illiterate patients or those who had any sensory, motor or communication disorder that would preclude them from responding to the test for detecting cognitive decline were excluded from the study. Patients who were moribund, on supportive and monitoring appliances and those who had undergone a surgery that interfered with their performance on the cognitive tests were also excluded. The family member living with the subject was interviewed as the key informant. Informed consent was taken from both the patients and the informants.

The Mini Mental State Examination (MMSE)^6^ was used to identify subjects with cognitive dysfunction. Presence of cognitive decline was defined using a cut-off score of 24 on the MMSE. Patients with a score of <24 out of 30 were categorized as having cognitive decline. Others were grouped as having normal cognition. Information on recent history of decline in memory, thinking and reasoning ability was elicited from the subject and the key informant using the informant and subjective versions of the Global Rating of Memory Decline (GRMD) and Global Rating of Intellectual Decline (GRID).^7^ The GRMD was used to assess if the patient had any problem with memory compared to the past (answered as Yes, Sometimes and No) and, if yes, whether the decline in memory interfered in any way with day-to-day life (answered as Yes, No, Do not know). The GRID was used to ask similar questions about any change in the patient's ability to think and reason.

The Statistical Package for Social Sciences was used for data analysis.^8^ Chi-square test, *t* test, and ANOVA and Pearson correlation analysis were done to identify factors that significantly differentiated patients with decline in cognition from those with normal cognition. Variables that significantly differentiated the groups at univariate analysis were entered as independent variables in a step-wise, two-group discriminant function analysis (Wilk lambda method) to delineate the variables that discriminated the patients with cognitive decline from those with normal cognition.

The mean age of patients was 70.5 years (SD±6.8). Twenty-five subjects (19%) were below 65 years of age, 77 (59%) between 65 and 74 years, 21 (16%) between 75 and 84 years and 7 (6%) were ≥85 years. Sixty-seven patients were admitted to the surgical unit and 63 to the medical unit. The duration of the medical/surgical illness was <2 weeks in 85 patients (65%), 2–4 weeks in 9 (7%) and >4 weeks in 36 patients (28%). The common surgical problems were general surgical conditions such as hernia, benign tumours in 34%, and orthopaedic conditions such as intervertebral disc problems, osteoarthritis and hip fractures in 36% of patients. The common medical diagnoses were cardiorespiratory conditions such as heart failure and obstructive pulmonary airway disease in 46% and infections such as gastroenteritis and urinary tract infections in 21% of patients. A neurological disorder such as stroke was diagnosed in only 5 out of 63 patients (8%).

## RESULTS

On the GRMD, subjective decline in memory was recorded in 71 patients, with 62 of them reporting that the decline interfered with their daily life. The corresponding number of informants reporting memory decline was 72 and 60. Subjective decline in intellectual function was scored on the GRID in 91 patients, with 55 of them reporting that it interfered with daily functioning. Intellectual decline in the patients was reported by 88 informants, with 55 of them reporting that it interfered with functioning.

The mean score on the MMSE was 23.5 (SD±5.7) with a minimum score of 4 and a maximum of 30. The age correlated negatively with MMSE scores (r= −0.319, 2-tailed p=0.000). Cognitive decline was diagnosed in 54 subjects (41.5%). Patients with cognitive decline were significantly older than those with normal cognition (72.3 years, SD±8.3 vs 69.2 years, SD±5; *t*=2.64, degrees of freedom [df]=128, p<0.01). Significantly more patients ≥70 years of age had cognitive decline compared with those ≤70 years of age (29 of 56 vs 25 of 74, chi-square=4.25, df=1, p<0.05). There was no significant difference between the two groups in the male to female ratio (37:17 for cognitive decline and 48:28 for normal cognition). The emergency room was more often the point of entry to the hospital for cases with cognitive decline (44 of 54) compared with 48 of 76 with normal cognition (chi-square=5.12, 2-tailed p<0.05). Significantly more patients admitted to the medical units had cognitive decline (33 of 63) compared with surgical admissions (21 of 67) (chi-square=5.92, df=1, 2-tailed p<0.05). The medical/surgical illness was more often acute (less than 2 weeks) in patients with cognitive decline (42 of 54) than in those with normal cognition (43 of 76) (chi-square=6.27, df=1, 2-tailed p<0.05). The discriminant function analysis showed that duration of illness and age discriminated significantly between those with and without cognitive decline (Wilk lambda=0.911, chi-square=11.878, df=2, p<0.01).

## DISCUSSION

The prevalence of cognitive decline as defined by MMSE in the hospitalized elderly population in this study was 41.5%. This rate of prevalence falls within the range described in the literature.^4^,^5^ There were a few patients in this study with a known neurological disorder. This indicated that cognitive decline was a common problem among elderly inpatients even if they had no known neurological disorder at the time of admission to hospital. Many patients and their relatives reported decline in memory and intellectual functioning before hospitalization. Patients with such decline were more often detected to have cognitive decline in the hospital. Thus information about the history from relatives could be useful in screening for cognitive decline in hospitalized elderly patients.

Saravay said that a hospital admission may be a brief but invaluable window of opportunity to identify and initiate treatment for a previously unrecognized mental disorder.^9^ The high prevalence of cognitive decline in elderly inpatients in several studies highlighted the need for physicians and surgeons to be aware of the condition in their patients. Screening to detect cognitive decline in elderly inpatients could facilitate investigation to identify reversible causes of dementia and plan appropriate treatment to improve the outcome.^10^ This study showed that patients ≥70 years of age with an acute medical problem were the most likely have cognitive problems. Elderly patients admitted through the emergency room may require special attention to screen for cognitive disturbances.
